# Chemical and mechanical patterning of tortoise skin scales occur in different regions of the head

**DOI:** 10.1016/j.isci.2025.112684

**Published:** 2025-06-04

**Authors:** Rory L. Cooper, Ebrahim Jahanbakhsh, Michel C. Milinkovitch

**Affiliations:** 1 Laboratory of Artificial & Natural Evolution (LANE), Department of Genetics & Evolution, University of Geneva, 1211 Geneva, Switzerland; 2 SIB Swiss Institute of Bioinformatics, Geneva, Switzerland

**Keywords:** Biological sciences, Zoology, Evolutionary biology

## Abstract

Vertebrate skin appendages are diverse micro-organs such as scales, feathers, and hair. These units typically develop from placodes, whose spatial patterning involves conserved chemical reaction-diffusion dynamics. Crocodile head scales are a spectacular exception to this paradigm, as they instead arise from a mechanically dominated process of compressive folding driven by constrained skin growth. Here, we reveal that chemical versus mechanical processes pattern tortoise scales in different regions of their head. Indeed, we show that placode-derived scales emerge across the peripheral head surfaces while remaining absent from the central dorsal region where scales subsequently form through a mechanical folding process. Using light sheet microscopy, we build a three-dimensional mechanical model that qualitatively recapitulates the diversity of scale patterns observed in this central head region in different tortoise species. Overall, our analyses indicate that mechanical head-scale patterning likely arose before the divergence between Testudinata and Archosauria, and was subsequently lost in birds.

## Highlights

• Systems of molecular interactions typically mediate skin appendage patterning
• Crocodile head scales instead self-organize from mechanical compressive skin folding
• Tortoises exhibit molecular and mechanical patterning in different regions of the head
• Mechanical head scale patterning arose in the ancestor of Testudinata and Archosauria

## Graphical abstract

**Figure:**
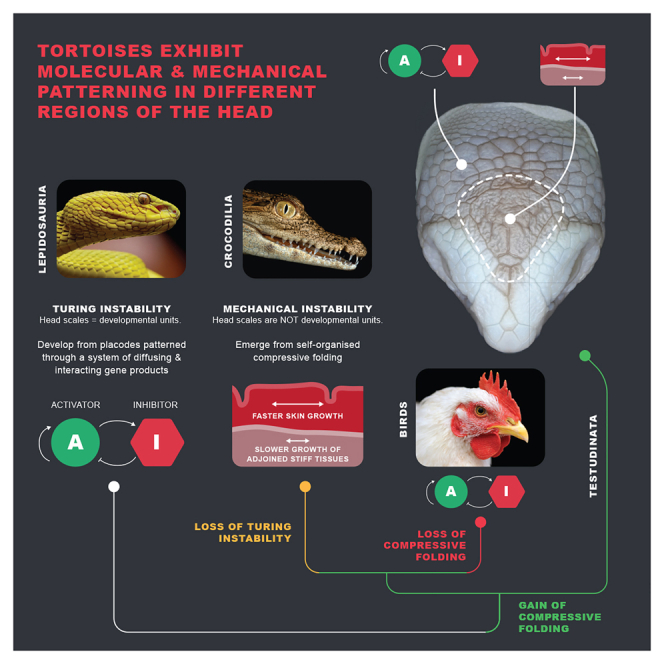

## Introduction

The placode-derived skin appendages adorning the vertebrate integument, such as scales, feathers, and hair, exhibit remarkable morphological and functional diversity. However, previous research has demonstrated that their spatial patterning is broadly underpinned by conserved developmental processes.^1^^,^^2^^,^^3^ This includes the chemical (i.e., molecular) reaction-diffusion (RD) patterning^4^^,^^5^^,^^6^^,^^7^^,^^8^ of placodes observed throughout phylogenetically distinct vertebrates, from the scales of sharks and snakes to the hair of mice.^9^^,^^10^^,^^11^^,^^12^ In these RD systems, the interactions among activatory and inhibitory morphogens produce a spotted pattern of molecular markers that form a template defining the spatial distribution of placodes. Hence, placodes, and the interactions of gene products that cause their emergence, constitute the conserved foundations of diverse skin appendages.^2^^,^^3^^,^^13^^,^^14^ Placodes are characterized by a local thickening of the epidermis, an associated aggregation of underlying dermal cells, and conserved epidermal and dermal gene-mediated signaling. Importantly, recent research has shown that mechanochemical systems, i.e., chemical systems with integrated mechanical cues, can also contribute to the patterning of skin appendage placodes. For example, the aggregation of dermal cells can mechanically compress the overlying epidermis, resulting in local signaling feedback associated with the patterning of feather follicles.^15^^,^^16^^,^^17^^,^^18^^,^^19^ Hence, skin appendages that arise from either chemical or mechanochemical RD systems constitute individual developmental units, as each prospective structure typically arises from a single placode.

Although chemical self-organizational morphogenesis is widely prevalent in vertebrates (e.g., ref. ^7^^,^^20^^,^^21^), developmental processes dominated by tissue mechanics also contribute to the diversity of biological patterning.^22^^,^^23^^,^^24^^,^^25^^,^^26^ For example, both gyrification of the human brain and villification of the human and chicken gut can be explained by a process of compressive stress-induced buckling and folding arising from constrained tissue growth.^23^^,^^26^ The patterns that emerge from such predominantly mechanical processes are mediated by the relative growth rates and/or material properties of constituent adherent tissue layers, rather than by intricate networks of molecular signaling. Clearly, in addition to chemical^9^^,^^10^^,^^11^^,^^12^ and mechanochemical^15^^,^^16^^,^^17^^,^^18^ patterning systems, purely mechanical processes can also mediate embryonic patterning.^23^^,^^26^

**Figure 1:**
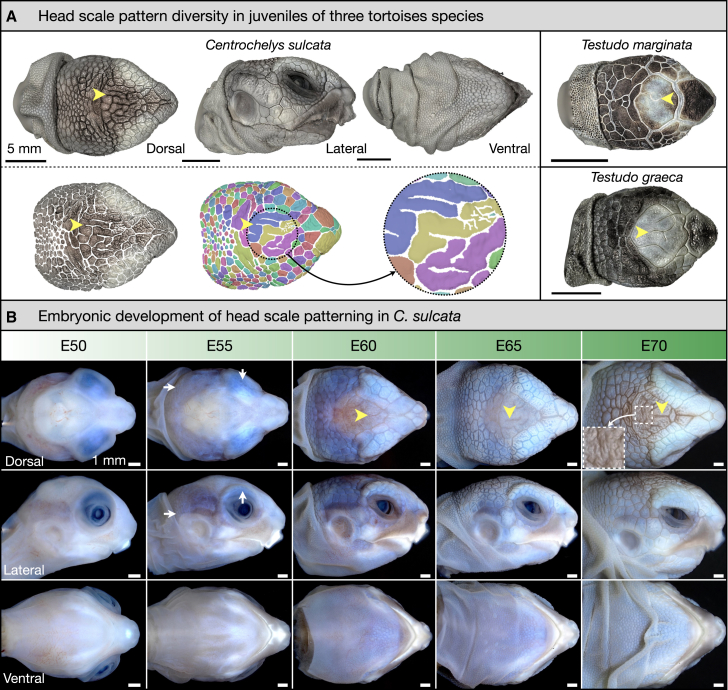
The diversity of tortoise head scale patterns (A) 3D reconstructions of juvenile tortoise heads from structured light scanning microscopy, including the sulcata tortoise (*Centrochelys sulcata*), the marginated tortoise (*Testudo marginata*), and the Greek tortoise (*Testudo graeca*). These meshes reveal that the central dorsal head surfaces exhibit signatures of mechanical patterning, including unjoined scale edges (yellow arrowheads).^22^^,^^27^ (B) Embryonic development of sulcata tortoise head scales. At E50, scales are absent. By E55, polygonal domains propagate across the peripheral dorsal head surface and lateral regions of the head (second column, white arrows). By E60, the head is mostly covered with polygonal scales, except for the central dorsal surface, where unjoined scale edges first become visible (yellow arrowhead). These unjoined scale edges continue to propagate from E65 to E70, giving rise to irregular polygons. By E70, the entire central dorsal head also exhibits fine-scale 3D geometry (inset).

Surprisingly, the development of crocodile head scales is dominated by such a mechanical patterning mechanism.^22^^,^^27^ Indeed, these non-overlapping polygonal scales are not individual developmental units but instead arise from compressive folding driven by skin growth that is frustrated by its attachment to the underlying tissues.^27^ Such a process does not seem to exist in squamate reptiles (lizards and snakes). But what about Testudinata (which include turtles, tortoises, terrapins, and their extinct relatives)? Previous research has shown that the scutes of tortoises develop from placodes which propagate in accordance with chemical RD dynamics.^28^ However, the developmental patterning of tortoise head scales has not, to our knowledge, previously been investigated. Strikingly, the central dorsal region of the tortoise head can exhibit scale domains that appear morphologically comparable to those of crocodile head scales, including signatures of mechanical patterning^22^^,^^27^ such as irregular polygonal geometries and unjoined scale edges (Figure 1). Therefore, we sought to investigate the embryonic patterning of tortoise head scales to elucidate which patterning processes contribute to their emergence.

**Figure 2:**
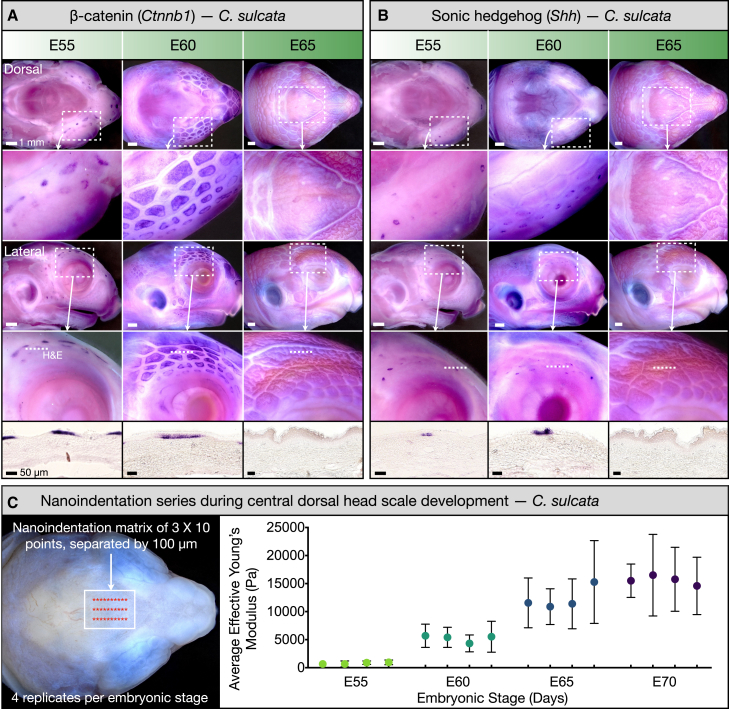
The development of sulcata tortoise head scales (A and B) WMISH reveals expression of the classic placode markers *Ctnnb1* (A) and *Shh* (B), localized to peripheral head scale primordia, from E55 to E60. Cryosections of these samples reveal the expression of *Shh* (B, bottom row) in a nested subregion of *Ctnnb1*-expressing epidermal cells (A, bottom row). By E65, cryosections of WMISH samples reveal a dense, keratinous, and undulating epidermis in the peripheral placode-derived region, lacking the local expression of *Ctnnb1* and *Shh* (A and B, bottom right panels) because patterning is completed. Conversely, placode-associated gene expression is never observed in the central dorsal head skin. (C) We use nanoindentation to examine changes in the skin surface stiffness of the embryonic sulcata tortoise head during the propagation of unjoined scale edges (C, left panel). We observed a rapid increase in effective Young’s modulus from E55 to E70 (C, right panel), revealing a substantial increase in skin surface stiffness in the central dorsal head surface. Mean values (±SD) are shown for each biological replicate.

**Figure 3:**
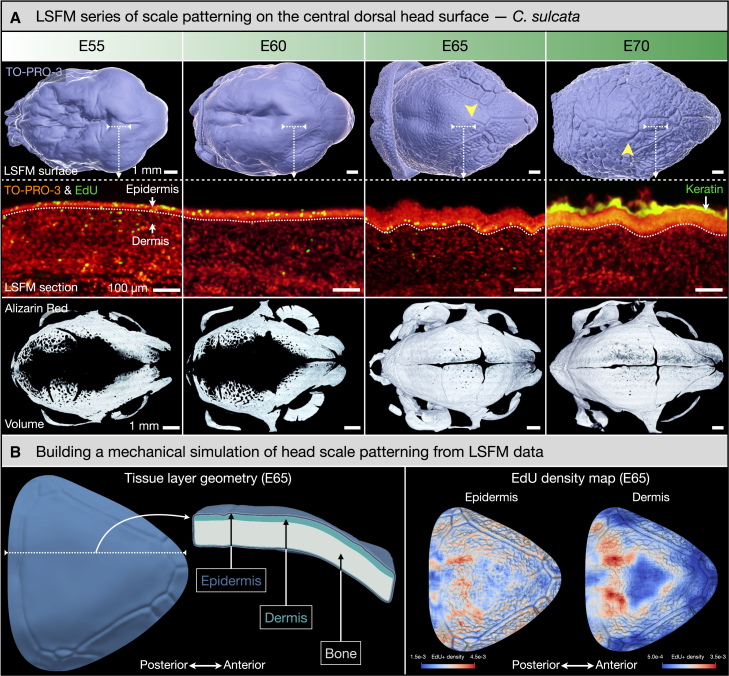
Building a mechanical model of tortoise central dorsal head scale patterning (A) We use LSFM to capture the precise tissue layer geometry of embryonic sulcata tortoise heads, from E55 until E70. Surface reconstructions from nuclear staining (with TO-PRO-3 Iodide; top row) show the emergence of unjoined scale edges (yellow arrowheads) and asymmetric scale domains on the dorsal head surface. Optical sections (middle row) reveal the rapid thickening and keratinization of the densely packed epidermis from E55 to E70, as well as the spatial distribution of proliferating cells (labeled with EdU) in the epidermis and dermis. Alizarin red staining reveals the progressive ossification of the skull (bottom row), revealing that the onset of skin surface patterning in the central dorsal head surface only occurs after the near-complete development of the skull at E65. (B) Left panel: we build a 3D finite-element numerical growth model integrating the tissue layers segmented from the LSFM data. Right panel: proliferating cell densities indicates somewhat homogeneous growth within the dermis and the epidermis (see also Figure S3), such that the numerical model assumes homogeneous growth within each layer but allows for different values between them (Table 1).

**Figure 4:**
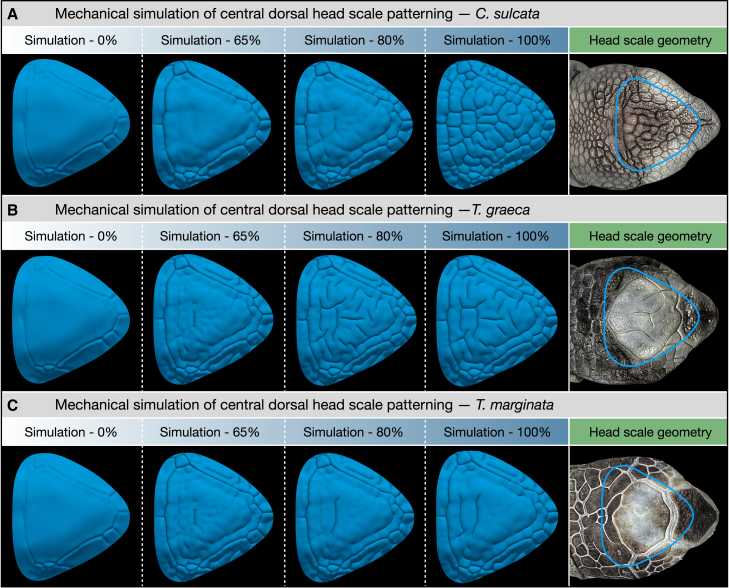
Our mechanical model recapitulates tortoise central dorsal head scale patterning (A–C) Variation of elastic and growth parameters (Table 1) allows us to qualitatively recapitulate the normal patterning of the central dorsal head region observed in sulcata tortoises ((A), see also Video S1), Greek tortoises ((B), see also Video S2) and marginated tortoises ((C), see also Video S3).

Here, we reveal that tortoise head scale development is mediated by sequential chemical versus mechanical patterning processes occurring in different regions of the head. Using whole mount *in situ* hybridization (WMISH), we demonstrate that the early developing polygonal scales covering the lateral and peripheral dorsal regions of the tortoise head exhibit the local expression of classic placode markers (Figures 2A and 2B). This indicates that these peripheral head scales are derived from placodes that are spatially organized through a chemical RD patterning system. Importantly, these placodes do not extend to the central dorsal head surface, i.e., the region in which we observe signatures of mechanical patterning. Using nanoindentation, we next quantify a rapid increase in tissue stiffness associated with the emergence of skin surface patterning in this central dorsal region (Figure 2C). We then acquire light sheet fluorescence microscopy (LSFM) data regarding the specific tissue layer geometries of the epidermis, dermis, and bone, and the distribution of cell proliferation within these layers (Figure 3A). Using these data, we build a 3D mechanical growth model (Figure 3B) and reveal that compressive stress associated with constrained skin growth is sufficient to qualitatively recapitulate the diverse head scale folding patterns observed in different tortoise species (Figure 4).

Although Testudinata has long been considered as the basal linage of amniotes (e.g., ref. ^29^), our phylogenomic analyses have indicated that they instead form the sister group to Archosauria^30^—i.e., a group of sauropsid tetrapods that include birds and crocodilians, as well as their extinct relatives (such as non-avian dinosaurs, pterosaurs and various marine reptiles). Note that this Testudines-Archosauria sister-group relationship was largely confirmed by subsequent studies (e.g., ref. ^31^^,^^32^^,^^33^). In the light of these phylogenetic relationships, our results demonstrate that mechanical head scale patterning is likely a shared derived character (a synapomorphy) that arose in the common ancestor of Archosauria and Testudinata and was subsequently lost in birds.

## Results

### Tortoise head scale pattern diversity

First, we examine the head scale pattern diversity of three tortoise species: the sulcata tortoise (*Centrochelys sulcata*; also called “African spurred tortoise”), the Greek tortoise (*Testudo graeca*), and the marginated tortoise (*Testudo marginata*) (Figure 1A). We present 3D surface meshes of juvenile tortoise heads acquired using structured light scanning microscopy. These tortoises all exhibit convex polygonal, non-overlapping scales on the peripheral regions of their dorsal head surfaces. Conversely, at the central dorsal head region, we observe diverse skin surface patterns which include unjoined edges (yellow arrowheads in Figure 1A). Segmenting isolated scale domains in the sulcata tortoise highlights the disorder of this highly asymmetrical network of edges (Figure 1A, bottom-left panels), a likely signature of mechanical patterning, comparable to what is observed on the face and jaws of the Nile crocodile.^22^^,^^27^

### Embryonic patterning of tortoise head scales

Next, we examine the emergence of sulcata tortoise head scale patterning during embryonic development (Figure 1B). At embryonic day 50 (E50), scales are absent from the entire head. However, by E55, polygonal units are observed propagating across the peripheral dorsal head surface and the lateral regions of the head (Figure 1B, second column, white arrows). By E60, these polygonal scales cover the entire head, except for the central dorsal head surface, in which unjoined scale edges first become visible (Figure 1B, third column, yellow arrowhead). From E65 to E70, the skin of the entire head undergoes rapid keratinization while unjoined scale edges continue to form and propagate on the central dorsal head surface (Figure 1B, fourth and fifth columns, yellow arrowheads) following dynamics reminiscent of the self-organized mechanical patterning of crocodile head scales. Note that by E70, the entire central dorsal head surface also exhibits fine-scale wrinkles (Figure 1B, fifth column, inset). Head scale emergence in the Greek tortoise follows a comparable trajectory, albeit during an earlier time window, due to the shorter total incubation period of this species (Figure S1A). Importantly, when we examine the intraspecific diversity of the central dorsal head surface in late-stage embryonic (E55) or juvenile tortoises of both sulcata and Greek tortoises (Figure S2), we observe the formation of highly variable head scale patterns, as would be expected from a mechanically driven system.^22^

To examine whether chemical RD patterning of conserved placodes can explain the emergence of tortoise head scales, we next used WMISH to visualize the expression of the classical placode markers, *β-catenin (Ctnnb1)* and sonic hedgehog (*Shh*), at multiple stages of sulcata head scale development (Figures 2A and 2B).^3^^,^^11^ At E55, we observe the epidermal expression of both *Ctnnb1* and *Shh* localized to peripheral head scale placodes (Figures 2A and 2B, left columns). By E60, polygonal head scale primordia propagate more extensively to cover both the peripheral dorsal head surface and the lateral head surface (Figures 2A and 2B, middle columns). Cryosections of samples at these two developmental stages (E55 and E60) reveal that these placodes express *Shh* in a nested subregion of *Ctnnb1*-expressing epidermis, bearing notable similarity to the expression patterns characterizing hair placodes in mammals,^34^ feather placodes in birds,^35^ body scales in crocodiles,^2^^,^^3^^,^^22^ as well as head and body scales in squamate reptiles.^3^ At E65, unjoined scale edges are visible in the central dorsal head surface but no local expression patterns of *Ctnnb1* or *Shh* are observed in this domain (Figures 2A and 2B, right columns). Therefore, a chemical RD patterning system does not appear to mediate the propagation of unjoined scale edges in the central region of the dorsal head surface. Local expression of *Ctnnb1* or *Shh* is also absent at that stage in skin cryosections performed in the adjacent peripheral region because chemical patterning is completed. Note that the fine undulations within the placode-derived scales bordering the central dorsal region may be indicative of the compressive wrinkling of the epidermis caused by its faster growth than the underlying adherent dermis (see also Figure 1B, fifth column, inset). Overall, these results demonstrate that although peripheral head scales of the sulcata tortoise express classic placode markers associated with chemical RD patterning, skin patterning of the central dorsal head surface is not associated with local gene signaling. Therefore, we next investigate whether a mechanical process can explain the patterning of this domain.

The patterns arising from mechanical instabilities are determined by the relative growth rates and material properties of the constituent tissue layers.^24^^,^^26^^,^^27^^,^^36^ Using nanoindentation, we quantified the stiffness (effective Young’s modulus (Pascals, Pa)) of the skin surface in the central dorsal head surface across multiple stages of the embryonic sulcata tortoise head skin (Figure 2C; see ‘STAR Methods’). We observe a rapid increase in effective Young’s modulus from E55 to E70, indicative of a substantial increase in skin surface stiffness during the propagation of unjoined scale edges in the central dorsal head. Nanoindentation also reveals comparable developmental changes in skin stiffness during the emergence of unjoined scale edges in the Greek tortoise (Figure S1B). Importantly, this stiffening of the skin surface is likely required to yield the patterns observed in the tortoise central dorsal head surface. Note, however, that these nanoindentation experiments only inform us on the large increase in skin surface stiffness (very likely due to increased keratinization of the epidermis) but do not provide information on changes in stiffness of the deeper dermal tissue.

### Building a mechanical growth model of head scale patterning

We recently quantitatively demonstrated that compressive stress resulting from skin growth that is frustrated by its attachment to the underlying stiff tissues, mediates the self-organized patterning of crocodile head scales.^27^ To investigate whether a comparable mechanical process can explain the patterning of the central dorsal head surface of tortoises, we next present a mechanical simulation built from 3D volumetric data acquired in sulcata tortoises using LSFM.

First, LSFM surface reconstructions of embryonic sulcata tortoise head samples labeled with nuclear staining (with TO-PRO-3 Iodide) illustrates the propagation of unjoined scale edges from E65 to E70 (Figure 3A, top row), as described above with images acquired with reflection light microscopy (Figure 1B). Importantly, nuclear staining also allows us to precisely and individually capture the geometry of the dermis and epidermis comprising the skin of the sulcata tortoise head because of the dramatic difference in cell density between these two tissue layers (Figure 3A, middle row). LSFM sections reveal a rapid increase in epidermal thickness from E55 to E70. Second, we used 5-ethynyl-2′-deoxyuridine (EdU) labeling and detection to visualize proliferating cells within the epidermis and dermis (Figure 3A, middle row; Figure S3). Third, we used Alizarin Red staining to capture the geometry of the developing skull underlying the epidermis and dermis (Figure 3A, bottom row). Ossification of the dorsal skull remains largely incomplete at E55 and E60. Surface reconstructions of samples at these two stages indicate the presence of a depression of the skin overlying this same region (Figure 3A, top row). We attribute this deformation to deep tissue shrinkage caused by dehydration during sample preparation, as there is no underlying bone layer present to support the skin. By E65, skull mineralization has dramatically advanced across the dorsal head. Importantly, E65 constitutes the onset of skin surface patterning in the central dorsal head. This observation is consistent with mechanical creasing of the skin that requires the presence of an adherent and stiffer underlying tissue characterized by less growth. In other words, the development of the underlying bone establishes a new boundary condition allowing for the mechanical creasing pattern to form in the dorsal region of the tortoise head.

Next, we build a finite-element (FE) 3D numerical growth model (see STAR Methods) integrating these LSFM data (Figure 3B) as described in our previous study.^27^ In brief, the 3D volumes of the two skin layers and underlying bone are represented as tetrahedral meshes, and deformation induced by growth is performed with finite-strain theory applied to the neo-Hookean material model. Note that a rather homogeneous growth *within* both skin layers (right panel of Figure 3B) is observed in the central dorsal area of the head. Using elastic and growth parameters (Young’s modulus, Poisson’s ratio, as well as normal and tangential growth; Table 1) similar to those optimized for Nile crocodile head scale patterning^27^ suffices to recapitulate the normal patterning of the central dorsal head region observed in sulcata tortoises (Figures 4A and Video S1): starting from a smooth geometry, the mechanical growth simulation produces surface folds which propagate and interconnect. In addition, manual variation of these parameters (Table 1) allow us to produce patterns that qualitatively resemble those of the two other species investigated here: the Greek and marginated tortoises (Figures 4B and 4C; Videos S2 and S3). Hence, our growth simulations successfully recapitulate the proposed mechanical-driven process of scale patterning observed in the central dorsal head region of multiple tortoise species.

**Table 1:** Mechanical model parameters used for skin folding simulations of three different species of tortoises

	$E_{epid}$	$E_{dermis}$	$\nu_{epid}$	$\nu_{dermis}$	$G_{T/N,epid}$	$G_{T/N,dermis}$
*C*. *sulcata* (Figure 4A)	3	1	0.1	0.4	0.6/0	0.6/0.3
*T*. *graeca* (Figure 4B)	2	1	0.25	0.45	0.5/0	0.7/0.1
*T*. *marginata* (Figure 4C)	2	1	0.25	0.45	0.45/0	0.7/0.1

These include Young’s modulus (E) and Poisson’s ratio (ν) as well as the final growth values in the tangential ($G_{T}$) and normal ($G_{N}$) directions for dermis and epidermis. Relative growth is normalised by considering the bone layer as a rigid and non-growing material.

Video S1. Mechanical growth simulations recapitulate sulcata tortoise central dorsal head scale patterning, see also Figure 4

Video S2. Mechanical growth simulations recapitulate Greek tortoise central dorsal head scale patterning, see also Figure 4

Video S3. Mechanical growth simulations recapitulate marginated tortoise central dorsal head scale patterning, see also Figure 4

## Discussion

Overall, our results demonstrate that the patterning of tortoise head scales occur sequentially via two different processes. First, placode-derived scale domains propagate across both the peripheral dorsal head surface and the lateral head surface (Figure 2). Second, compressive stress derived from skin growth, that is frustrated by the attachment of the skin to the underlying stiff tissues, produces skin creasing in the form of unjoined scale edges and asymmetric scale domains in the central dorsal head surface (Figures 3 and 4). In concurrence with previous research,^22^^,^^23^^,^^26^^,^^27^^,^^36^ this demonstrates that biological patterning can be mediated by purely mechanical processes, in addition to chemical^9^^,^^10^^,^^11^^,^^12^ and integrated mechanochemical systems.^15^^,^^16^^,^^17^^,^^18^ Note that, because the length scale of a folding pattern is proportional to the thickness of the folding tissue, the fine-scale wrinkling observed at late stages on the entire central dorsal head surface (Figure 1B, fifth column, inset) cannot be caused by the same process as the creasing of the whole skin. Hence, this fine pattern is likely caused by the wrinkling of the thin epidermis growing, at late stages, on the adherent underlying dermis.

The finite-element 3D numerical growth model we are using here does not allow for tissue plasticity as it relies on finite-strain theory implementing the neo-Hookean hyperelastic model —known to behave appropriately for many soft biological tissues under small to large deformations (see STAR Methods). Although we cannot exclude that some plasticity (flow) occurs, the self-organized mechanical patterning of scales on the face and jaws of crocodiles,^27^ as well as on the top of the head in tortoises (as shown in this study), seem to be dominated by an elastic regime as these patterns are recapitulated under the neo-Hookean model. Implementation of a more complex visco-elastic model (combining elastic, plastic and yield properties) might be required for recapitulating other morphogenetic processes.

Although a given set of dermis versus epidermis mechanical parameters (tissue growth, thickness and stiffness) tends to robustly generate a given type of pattern, our investigation of the self-organized patterning of crocodile head scales indicates that small differences of boundary conditions, and of mean parameter values between individuals, as well as stochastic spatial fluctuations affecting these parameters across the skin of any individual, explain that the exact number and positions of edges (folds) is not only variable among individuals but also between the left and right sides of the face.^22^^,^^27^ Hence, different individual crocodiles of a given species can be easily distinguished by their unique pattern (a ‘faceprint’ akin a fingerprint). Still, all members of a given species will exhibit patterns that are statistically similar (e.g., in terms of pattern length scale and approximate number of polygonal domains). On the other hand, head scale pattern statistics differ substantially among crocodilian species. For example, the typical head scale pattern of American alligators exhibits much less edges than does that of Nile crocodiles, whereas the latter shows less edges (hence, less numerous and larger polygonal scales) than spectacled caimans. A mechanical morphospace of skin folding has indicated that these differences are explained by interspecies variation of the relative epidermal versus dermal growth rates and elastic moduli.^27^

Here, we show that similar conclusions can be drawn for the mechanical patterning of scales on the top of the head of tortoises: the pattern differs among individuals within a species (Figure S2), but the variation is greater *among* species. Indeed, we observe that the sulcata tortoise exhibits substantially more folding in its central dorsal head surface than does the Greek tortoise, while the marginated tortoise shows even less folding (Figure 1A). Similarly to the situation observed for crocodilians, the evolution of tissue layer-specific growth rates and material properties is likely to have produced this diversity among distinct tortoise species (Figure 4; Table 1). The corollary to these variabilities is that the scales on the face and jaws of crocodiles, as well as skin features on the top of the head in tortoises, cannot be used as individual well-defined elements for species identification, whereas statistical properties of the pattern can. However, in the case of tortoises, statistical features of head scale patterns might be more difficult to use for species identification than diagnostic characters elsewhere on the body. For example, Greek tortoises generally exhibit an undivided supracaudal scute on the carapace, as well as spurs (small tubercles) on the thighs, whereas marginated tortoises exhibit a divided supracaudal scute and no spurs on the hind legs.

**Figure 5:**
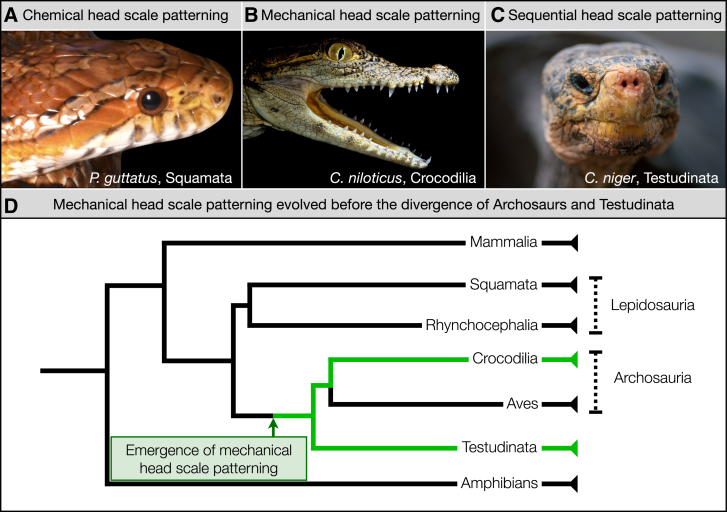
The developmental diversity of reptilian head scale patterning Reptilian head scales can arise in accordance with one of three processes: (A) head scales can emerge from individual placodes patterned via paradigmatic chemical RD as observed in snakes (here, the corn snake, *Pantherophis guttatus*); (B) head scales can also arise from a purely mechanical process^22^ of compressive folding driven by frustrated skin growth, as observed in crocodilians (here, the Nile crocodile, *Crocodylus niloticus*)^27^; or (C) head scales can emerge from sequential chemical and mechanical processes occurring in different regions of the head, as observed in tortoises (here, a Galápagos giant tortoise, *Chelonoidis niger*). In this latter process, the peripheral scales first emerge from RD-patterned placodes before compressive folding gives rise to skin surface patterning in the central dorsal head surface. (D) As mechanical patterning processes contribute to the head scale patterning of both crocodiles^22^^,^^27^ and tortoises (this study), mechanical compressive (growth-driven) head scale patterning is likely a synapomorphic trait that arose before the divergence between Archosaurs and Testudinata, and was subsequently lost in birds (Aves).

Our findings also have important implications regarding the macroevolution of the distinct developmental mechanisms underpinning reptilian scale patterning. Reptilian body and head scales typically arise from individual placodes whose spatial distribution is patterned via chemical RD (possibly in conjunction with positional information on the head), as observed in the corn snake (*Pantherophis guttatus*)^12^ (Figure 5A). We previously demonstrated that crocodilian head scales provide a fascinating exception to this paradigm,^22^ as they instead arise from a mechanical process of compressive folding driven by skin growth that is frustrated by its attachment to stiff underlying tissues (Figure 5B).^27^ Interestingly, tortoise head scales exhibit sequentially occurring chemical and mechanical patterning processes, with the peripheral head scales first arising from placodes, before skin folding in the central dorsal head surface subsequently emerges from compressive stress (Figure 5C). Therefore, a comparable mechanical developmental process contributes to the head scale patterning of both tortoises and crocodiles. Because Testudinata are the sister group of Archosaurs,^30^^,^^31^^,^^32^^,^^33^ the parsimony argument prompts us to suggest that mechanical compressive head scale patterning is a synapomorphic trait that arose before the divergence between these two lineages and was subsequently lost in birds (Figure 5D).

### Limitations of the study

Unlike our previous study investigating the mechanical patterning of crocodile head scales,^27^ here, we were unable to experimentally perturb the development of tortoise head scales. There are two main reasons for this. First, tortoise embryos exhibit dramatic developmental variability, even within the same clutch, such that it is difficult to reproducibly obtain specific developmental stages and corresponding experimental results. Second, the skin of the tortoise’s head is much more keratinized at the onset of folding relative to the crocodile, and is therefore much stiffer (Figure 2C). Consequently, we could attempt pharmacological perturbations only in a very restricted number of embryos of variable stages and the very stiff skin was more resistant to ectopic folding induced by experimentally exacerbated proliferation. Despite this, our other results provide strong evidence for the mechanical folding of skin on the dorsal head surface of tortoises.

## Resource availability

### Lead contact

Further information and requests for resources and reagents should be directed to, and will be fulfilled by, the lead contact, Michel C. Milinkovitch (michel.Milinkovitch@unige.ch).

### Materials availability

Further information and requests for resources and reagents should be directed to, and will be fulfilled by, the lead contact.

### Data and code availability

• All data needed to evaluate the conclusions in the paper are presented in the paper and/or the supporting information.
• The executables and original code used for the numerical simulations presented here are provided as a public Code Ocean capsule at https://codeocean.com/capsule/7775635/tree/v1.
• Any additional information required to re-analyze the data reported in this paper is available from the lead contact author upon request.

## Acknowledgments

We thank A. Debry and F. Montange for technical assistance with tortoise husbandry. We thank Oliver Antonini for providing photos of sulcata juveniles. Additionally, we thank C. Langrez and A. Tzika for undertaking *in situ* hybridization probe design.

This work was supported by grants to M.C.M. from the 10.13039/501100001711Swiss National Science Foundation (FNSNF; grant 10001627), the International Human Frontier Science Program Organisation (HFSP RGP0019/2017), and the 10.13039/501100000781European Research Council (ERC; advanced grant EVOMORPHYS) under the 10.13039/501100007601European Union’s Horizon 2020 research and innovation program. The funding bodies played no role in the design of the study, collection, analysis, and interpretation of data and in writing the manuscript.

## Author contributions

Conceptualization: M.C.M. Methodology: R.L.C., E.J., and M.C.M. Investigation: R.L.C., E.J., and M.C.M. Visualization: R.L.C., E.J., and M.C.M. Supervision: M.C.M. Writing, review and editing: M.C.M., R.L.C., and E.J.

## Declaration of interests

The authors declare that they have no competing interests.

## STAR★Methods

### Key resources table

**Table:** 

REAGENT or RESOURCE	SOURCE	IDENTIFIER
**Biological samples**		

Tortoise embryos	Tropiquarium de Servion’ (Servion, Switzerland)	N/A
	Erlebnis-Bauernhof Wannenwis (Waldkirch, Switzerland)	N/A
	Breeding colony of the Milinkovitch-Tzika laboratory (University of Geneva, Switzerland)	N/A

**Chemicals, peptides, and recombinant proteins**		

Tween 20	SigmaAldrich	P1379
Triton X-100	SigmaAldrich	93443
Proteinase K	SigmaAldrich	P6556
DIG RNA labeling kit	Roche	11175025910
Anti-Digoxigenin-AP, Fab fragments	Roche	11093274910
TO-PRO-3 Iodide	ThermoFisher	T3605
YO-PRO-1 Iodide	ThermoFisher	Y3603
Alizarin Red	ThermoFisher	400481000
Baseclick EdU Assay	Baseclick	BCK-EdU555IM100
Dichloromethane (DCM)	SigmaAldrich	270997
Dibenzyl ether (DBE)	SigmaAldrich	33630

**Software and algorithms**		

Imaris	https://imaris.oxinst.com/imaris-viewer	N/A
Meshlab	https://www.meshlab.net/	N/A
Matlab	https://www.mathworks.com/products/matlab.html	N/A
CUDA® C++ Core Libraries	https://github.com/NVIDIA/cccl	N/A

### Experimental model and subject details

Fertilised sulcata tortoise eggs were acquired from the ‘Tropiquarium de Servion’ (Servion, Switzerland) and Erlebnis-Bauernhof Wannenwis (Waldkirch, Switzerland). Greek tortoise eggs were acquired from our own breeding colony (Milinkovitch-Tzika laboratory, University of Geneva, Switzerland). All eggs were incubated at 31°C in moist vermiculite. Tortoise samples were fixed at the appropriate embryonic stages, i.e., those relating to head scale development (which varied between different species), and stored in 10% neutral buffered formalin (NBF) at 4°C. Maintenance of, and experiments with, all tortoise embryos and newborns were approved by the Geneva Canton ethical regulatory authority (authorization GE244) and performed according to Swiss law. These guidelines meet international standards.

### Method details

#### Structured light scanning microscopy and reflective light microscopy

Juvenile tortoise heads were scanned with an optical profilometer (Keyence VR) to create the 3D surface meshes shown in Figure 1A. Samples were scanned from multiple angles at 45-degree increments and individual meshes were stitched together using MeshLab. Individual head scales of the Sulcata tortoise were also manually segmented (Figure 1A, bottom panel) in MeshLab. Embryos in Figure 1B and Figure S1 were imaged with a Keyence VHX 7000 digital microscope.

#### Whole mount *in situ* hybridisation (WMISH)

WMISH of embryonic tortoise samples was undertaken as previously described.^37^ Samples were fixed overnight in 4% PFA, dehydrated into methanol (MeOH), bleached with 30% hydrogen peroxide in MeOH, prior to rehydration into PBS with Tween 20 (PBST) and permeabilisation with Proteinase K (Sigma-Aldrich). Samples were then hybridised overnight with an antisense digoxigenin (DIG)-labelled RNA probe. The following day, post hybridisation washes were undertaken with saline sodium citrate (SSC) buffer. Samples were then washed in blocking solution prior to overnight labelling with anti-DIG (Sigma-Aldrich). Next, samples were washed with six one-hour washes in Tris-buffered saline with Tween 20 (TBST). Finally, samples were washed in NTMT, before the colour reaction took place using 5-Bromo-4-chloro-3-indolyl phosphate (BCIP) and Nitroblue tetrazolium (NBT) in NTMT. Following WMISH, samples were post-fixed in 4% PFA and imaged with a Keyence VHX 7000 digital microscope. Next, samples were embedded in optimal cutting temperature (OCT) compound, sectioned with a cryostat (Leica CM1850), and imaged with an automated slide scanner (3DHISTECH).

#### Nanoindentation

A nanoindentor (Piuma, Optics11) was used to acquire stiffness measurements (Effective Young’s Modulus, Pascals (Pa)) from the dorsal head surface of a developmental series of *C*. *sulcata* (Figure 2) and *T*. *graeca* (Figure S1) samples. Fresh embryonic tortoise heads were dissected, positioned dorsal side upwards in a Petri dish, and submerged in PBS. Four biological replicates were used for each developmental stage. Samples were indented at a depth of 2 μm using a probe with a stiffness rating of 0.54 N/m and a tip radius of 99 μm. Each sulcata tortoise sample was indented 30 times in a 3 X 10 grid, whereas Greek tortoise samples were each indented 9 times in a 3 X 3 grid. Only load-displacement curves with a Hertizian contact fit model of ≥95% were subsequently analysed.

#### Light sheet fluorescence microscopy

Fixed tortoise heads were dehydrated into MeOH, bleached with hydrogen peroxide, and rehydrated into phosphate-buffered saline with Triton X-100 (Sigma-Aldrich) (PBST). Samples were incubated in either YO-PRO-1 or TO-PRO-3 Iodide (3:1000, ThermoFisher) for six hours to label cell nuclei. At collection, tortoise embryos were treated with an injection of EdU to label proliferating cells (Baseclick); embryo collection and fixation were undertaken 3 h after EdU injection. EdU-labelled cells were also detected following the Baseclick EdU detection kit guidelines. The calcified bone matrix of the skull was stained using Alizarin Red dissolved in potassium hydroxide (KOH). All samples were cleared following the iDISCO+ protocol.^38^ This involved dehydration into MeOH, prior to washing in 2/3 dichloromethane (DCM) with 1/3 MeOH for 3 h, washing in DCM for 1 h before multiple washes in dibenzyl ether (DBE) until fully transparent. Imaging was undertaken using a light sheet microscope (Ultramicroscope Blaze, Miltenyi Biotec). Image stacks were visualised using Imaris (Oxford Instruments). A minimum of three biological replicates were prepared and imaged at each developmental stage.

#### Segmentation of LSFM data

Using TO-PRO-3, EdU, and Alizarin Red, we segmented the light-sheet microscopy data to extract the geometry of the epidermis, dermis and bone tissues, as well as the distribution of proliferating cells in the two former. More specifically, the 3D images generated by LSFM on the basis of the TO-PRO-3 fluorescence signal were subjected to 3D Canny’s edge detection^39^ in MATLAB-R2021a, generating 3D binary images in which nonzero voxels form point clouds corresponding to two 3D surfaces: the surface of the epidermis and the epidermis-dermis boundary. For each of these two surfaces, we compute at each point the surface normal vector from the intensity gradient. The position of points and their corresponding normal vectors are then fed to a screened Poisson surface reconstruction algorithm^40^ in Meshlab^41^ to reconstruct triangular surface meshes, which effectively represent the initial point clouds in a much lighter format. The epidermis surface and the epidermis-dermis boundaries allow for computing the epidermis thickness across the investigated region. These segmented data were then used to build a finite element model of the tortoise central dorsal head skin.

For the segmentation of proliferating cells, we use a 3D principal curvatures approach^42^ on the EdU labeling fluorescence signal in the central dorsal head skin of an embryonic sulcata tortoise at E65, i.e., at the onset of head skin folding. This approach is highly efficient for individually segmenting cells when they are grouped (i.e., in contact). Additional details are given in our previous study on crocodiles.^27^ As these analyses indicate a rather homogeneous growth *within* each layer (Figure 3B, right panel), but different values between them, these two values are introduced in the numerical model (Table 1). For segmenting bone tissue, we use the 3D Canny’s edge detection of the Alizarin Red signal.

### Quantification and statistical analysis

#### Biomechanical model and numerical simulations

We use the segmented data to build a finite-element (FE) 3D numerical growth model. Triangular meshes were generated at the surface boundaries of the epidermis, dermis and bone (left panel of Figure 3B, and see previous section). The epidermis surface and the epidermis-dermis interface were smoothed to remove any artificial local deformations associated with sample preparation, including dehydration into methanol. The 3D volume of each of the three layers was represented as a tetrahedral mesh generated with TetGen.^43^ During simulated growth, the deformation of tetrahedral elements is realised through finite-strain theory with the neo-Hookean material model, known to behave appropriately under large deformations.^23^^,^^26^^,^^27^ Much additional details are given in our previous study on crocodiles.^27^ Note that the absolute values of stiffness (Young’s modulus) are irrelevant in the numerical simulations as the model key parameters are the relative dermis *versus* epidermis moduli.

To perform numerical simulations, the mechanical model is discretised for tetrahedral elements using the FE model and integrated with contact and viscous forces. The final model is then implemented in an in-house application that exploits NVIDIA® GPUs for high performance computation. For that purpose, we used the CUDA® programming language to develop intensive-computation kernels, whereas C++ is used for data management, geometry processing, input/output operations and the graphical user interface. The simulation input is a tetrahedral mesh that defines the geometry of the tortoise central dorsal head region (epidermis, dermis and bone layers). We include, both for epidermis and dermis, the Young’s modulus and Poisson’s ratio, the growth functions for each of the two layers. The deformation of the skin is then computed and the final geometry is generated as a tetrahedral mesh. Much additional information on the numerical model are available in our previous work on crocodiles.^27^
