# Supplementary material for: Chemical and mechanical patterning of tortoise skin scales occur in different regions of the head
Source: iScience. 2025 Jun 4;28(6):112684. doi: 10.1016/j.isci.2025.112684 (PMC12225933; doi:10.1016/j.isci.2025.112684)
Supplement: Document S1. Figures S1–S3 [file mmc1.pdf]

iScience, Volume *28*

Supplemental information

Chemical and mechanical patterning of tortoise skin scales occur in different regions of the head

Rory L. Cooper, Ebrahim Jahanbakhsh, and Michel C. Milinkovitch

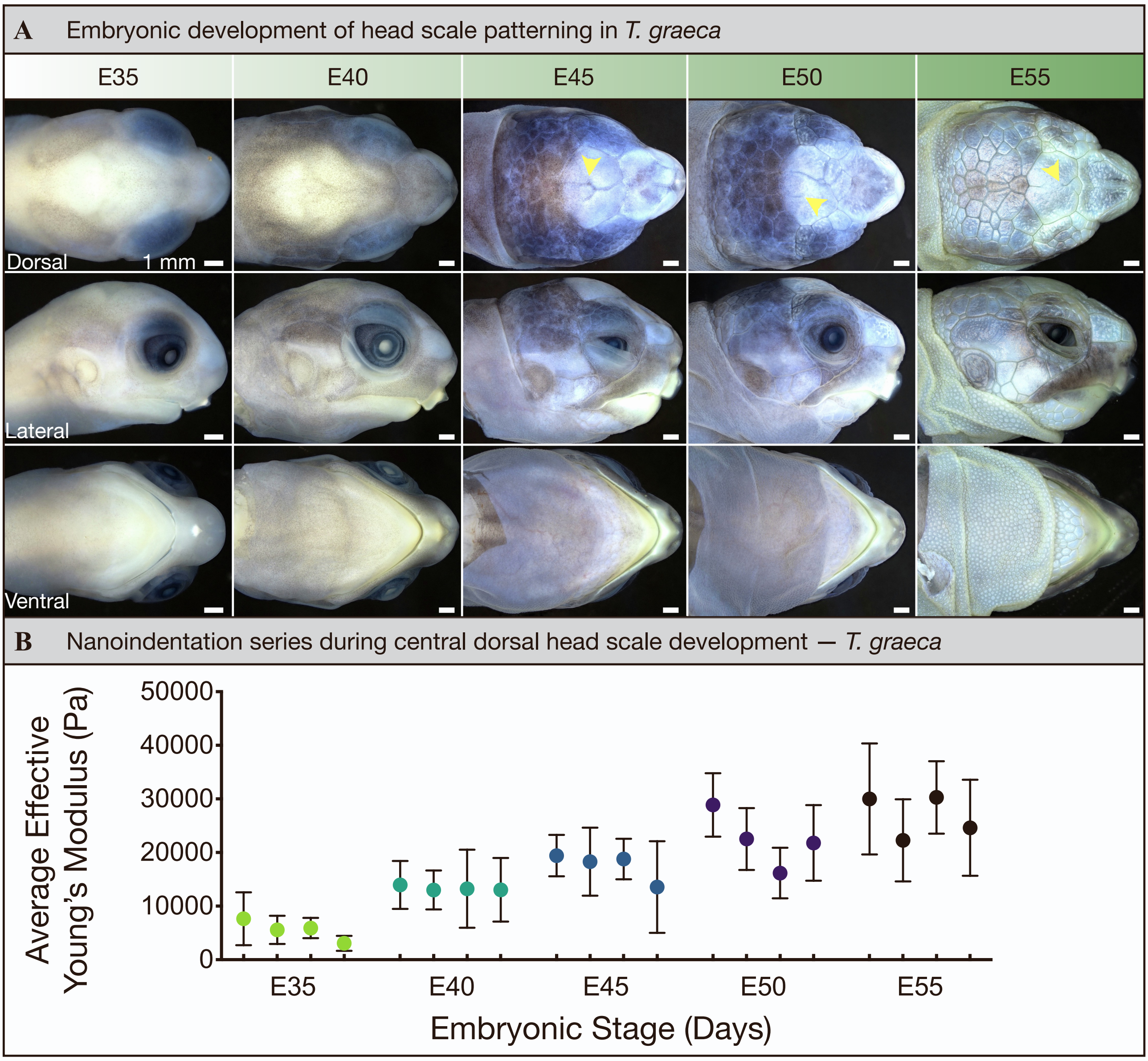

Figure S1 - Head scale patterning in the Greek tortoise (*Testudo graeca*). (**A**) Head scale patterning of the Greek tortoise follows a comparable trajectory to the head scale patterning of the sulcata tortoise (Fig. 1B). At E35, the tortoise head is smooth. Peripheral scale units first emerge from E40 onwards. By E45, the head is well covered with scales, and unjoined scale edges are observed propagating across the central dorsal head surface (yellow arrowheads). These unjoined scale edges continue to propagate until E55, which is close to the time of hatching. (**B**) Nanoindentation reveals a comparable increase in tissue stiffness in the central region of the tortoise head surface, as observed in the sulcata tortoise (Fig. 2B). Mean values (±SD) are shown for each biological replicate.

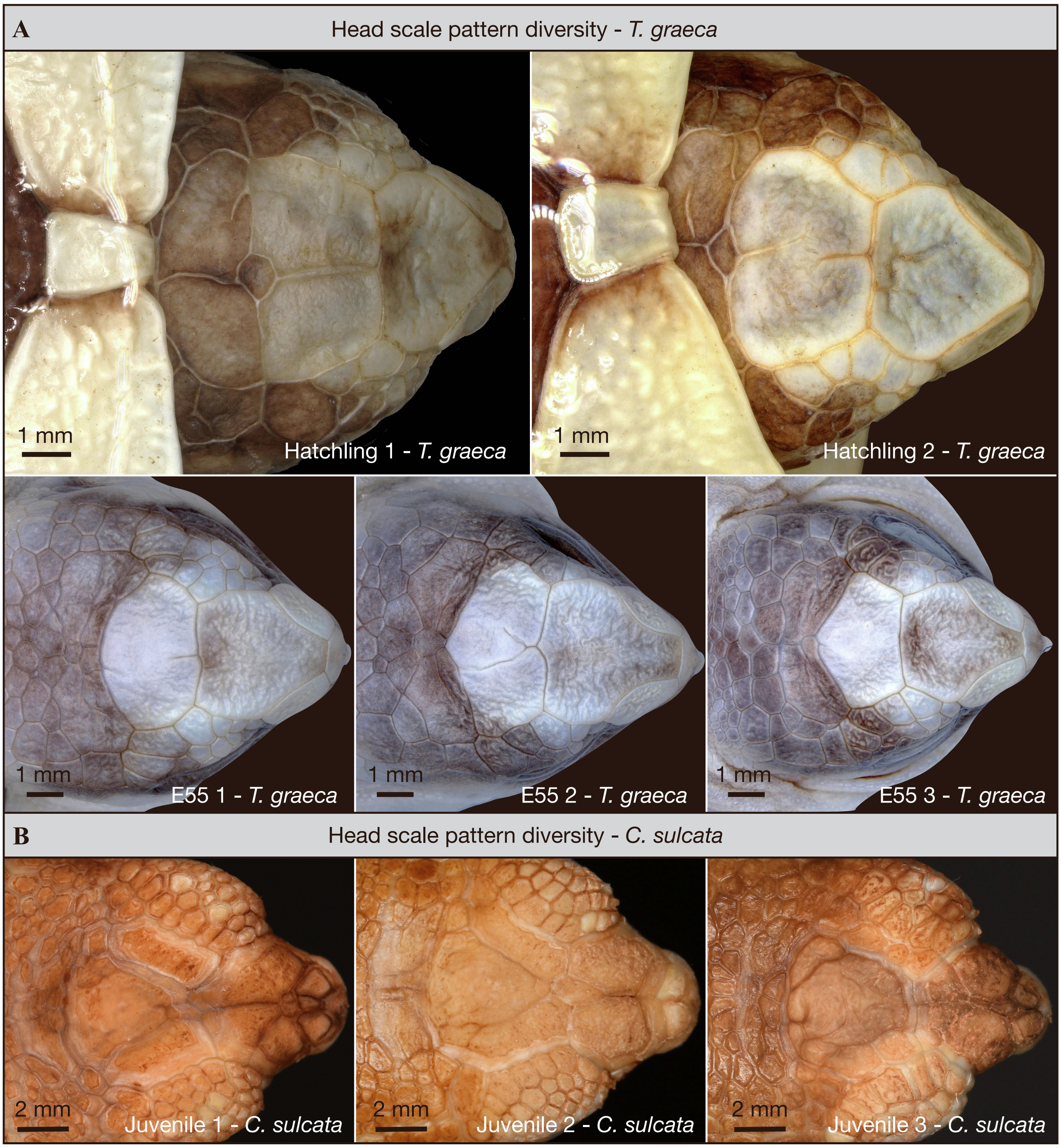

Figure S2 - Intraspecific tortoise head scale pattern diversity. We show the dorsal head skin surface patterning in (**A**) the Greek tortoise (two hatchlings in upper panels and three near-hatching embryos in lower panels) and (**B**) the sulcata tortoise (three six-month juveniles). These replicates highlight the variability of head scale patterns observed among individuals within a single tortoise species.

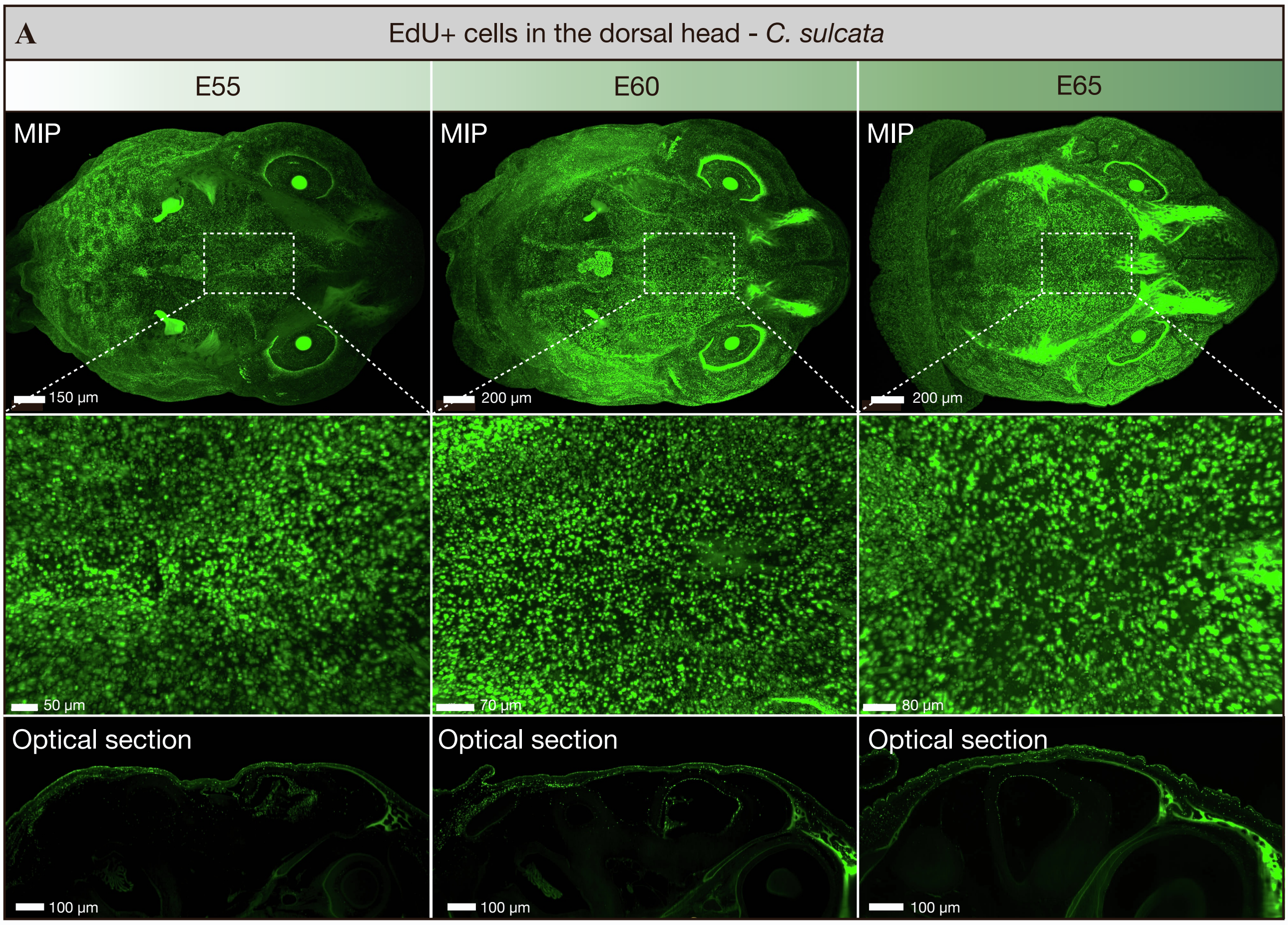

Figure S3 - EdU+ cells in the dorsal head of the Sulcata tortoise (*C. sulcata*). Labelling and detection of EdU+ cells using LSFM reveal substantial proliferation in the skin of the dorsal head of the Sulcata tortoise, from E55 to E65 (MIP = maximum intensity projection).
